# Management of transmodiolar and transmacular cochleovestibular schwannomas with and without cochlear implantation

**DOI:** 10.1007/s00106-020-00919-9

**Published:** 2020-10-12

**Authors:** S. K. Plontke, P. Caye-Thomasen, C. Strauss, S. Kösling, G. Götze, U. Siebolts, D. Vordermark, L. Wagner, L. Fröhlich, T. Rahne

**Affiliations:** 1 Department of Otorhinolaryngology, Head & Neck Surgery, Martin Luther University Halle-Wittenberg, University Medicine Halle, Ernst-Grube-Str. 40, 06120 Halle (Saale), Germany; 2 Department of Oto-Rhino-Laryngology, Head and Neck Surgery, Copenhagen University Hospital Rigshospitalet, Copenhagen, Denmark; 3 Department of Neurosurgery, Martin Luther University Halle-Wittenberg, University Medicine Halle, Halle, Germany; 4 Department of Radiation Medicine, Clinic for Radiology, Martin Luther University Halle-Wittenberg, University Medicine Halle, Halle, Germany; 5 Institute of Pathology, Martin Luther University Halle-Wittenberg, University Medicine Halle, Halle, Germany; 6 Department of Radiation Medicine, Clinic for Radiotherapy, Martin Luther University Halle-Wittenberg, University Medicine Halle, Halle, Germany

**Keywords:** Evoked potentials, auditory, brain stem, Schwannoma, intralabyrinthine, Neuroma, acoustic, Ear, inner, Rehabilitation

## Abstract

**Introduction:**

Hearing rehabilitation with cochlear implants has attracted increasing interest also for patients with cochleovestibular schwannoma. The authors report their experience with the surgical management of tumors with rare transmodiolar or transmacular extension and outcomes after cochlear implantation (CI).

**Methods:**

This retrospective case series included nine patients with either primary intralabyrinthine tumors or secondary invasion of the inner ear from the internal auditory canal. The primary endpoint with CI, performed in six patients, was word recognition score at 65 dB SPL (sound pressure level). Secondary endpoints were intra- and postoperative electrophysiological parameters, impedance measures, the presence of a wave V in the electrically evoked (via the CI) auditory brainstem responses, the specifics of postoperative CI programming, and adverse events.

**Results:**

Hearing rehabilitation with CI in cases of transmodiolar tumor growth could be achieved only with incomplete tumor removal, whereas tumors with transmacular growth could be completely removed. All six patients with CI had good word recognition scores for numbers in quiet conditions (80–100% at 65 dB SPL, not later than 6 to 12 months post CI activation). Four of these six patients achieved good to very good results for monosyllabic words within 1–36 months (65–85% at 65 dB SPL). The two other patients, however, had low scores for monosyllables at 6 months (25 and 15% at 65 dB SPL, respectively) with worsening of results thereafter.

**Conclusions:**

Cochleovestibular schwannomas with transmodiolar and transmacular extension represent a rare entity with specific management requirements. Hearing rehabilitation with CI is a principal option in these patients.

**Video online:**

The online version of this article (10.1007/s00106-020-00919-9) includes a video (2D and 3D versions) of the described surgical technique. Article and supplementary material are available at www.springermedizin.de. Please enter the title of the article in the search field, the supplementary material can be found under “Ergänzende Inhalte”.

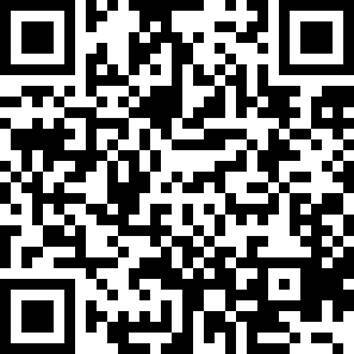

## Introduction

Cochleovestibular schwannomas are benign tumors of the eighth cranial nerve with an incidence of approximately 3.4/10^5^ [41]. They usually originate from the vestibular nerve and occur as unilateral, sporadic, nonsyndromic tumors. Bilateral occurrence has been observed in 5–10% of cases associated with neurofibromatosis 2 (NF2) [12]. The usual tumor locations are the internal auditory canal (IAC) and the cerebellopontine angle (CPA) [27]; however, they can also occur as intralabyrinthine schwannomas (ILS) in the terminal branches of the eighth cranial nerve in the inner ear (recently reviewed in [8]).

Hearing loss because of vestibular schwannomas (VS) negatively influences the quality of life and patients with this condition are increasingly interested in hearing rehabilitation with cochlear implantation (CI) [25, 28, 57]. Several case reports, case series and first systematic reviews have illustrated that after surgical removal of sporadic VS or VS associated with NF2, CI can lead to good hearing results, even though not all patients will achieve good speech understanding. The CI may be performed in one stage with tumor removal [3, 45, 50, 57] or in a second surgery [3, 4, 17, 22, 42]. Similar initial results for hearing rehabilitation are available for CI after radiotherapy [9, 28, 31] and with a wait and test and scan strategy [5]. For ILS limited to the inner ear, surprisingly good results with CI with respect to speech understanding have been reported, despite substantial cochlear trauma from the surgical tumor removal [2, 35, 36, 38].

**Fig. 1:**
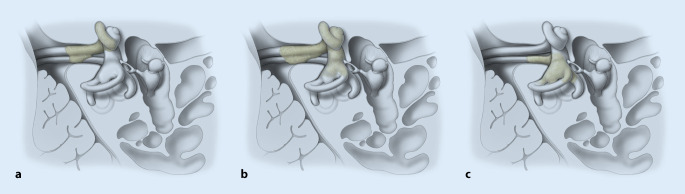
Schematic drawings of tumor extensions from the inner ear to the internal auditory canal. **a** Intracochlear schwannoma with transmodiolar extension. **b** Intravestibulocochlear schwannoma with transmodiolar extension. **c** Intravestibular schwannoma (+ partially semicircular canals) with transmacular extension. Additional growth into the cerebellopontine angle is also possible (not shown). The facial nerve is located cranially to the cochlear nerve and thus not shown. For simplification, only one vestibular nerve is shown. (Artist: Hans Jörg Schütze, Medical Illustrator, Köln, Germany)

Special subtypes of cochleovestibular schwannomas are located in the inner ear and the IAC, and sometimes the CPA, i.e., transmodiolar (TMOD, cochlea and IAC), transmacular (TMAC, vestibule and IAC), or translabyrinthine (TLab, intravestibulocochlear with TMOD and TMAC growth into the IAC) (Table 1, Fig. 1). For TMOD and TLab schwannomas, CI is possible (and meaningful) only with incomplete tumor removal because complete removal would also include destruction of the spiral ganglion cells in the modiolus, which are necessary for electrical stimulation through the implant. First results have been promising in patients for whom hearing rehabilitation was a high priority, including CI without intracochlear tumor removal [6] or with partial removal of the intracochlear parts of the tumor and reduction of the retrocochlear portion only (IAC + CPA) [39].

**Table 1:** Classification of cochleovestibular schwannomas with location in the inner ear and the internal auditory canal. (After Kennedy et al. [21] and van Abel et al. [54])

Classification	Abbreviation	Cochlea	Vestibule ± SCC	IAC	Middle ear	CPA
Transmodiolar^a^	TMOD	X	(X)	X	–	–
Transmacular^b^	TMAC	–	X	X	–	–
Translabyrinthine^c^	TLab	X	X	X	–	–
Transotic	TO	X	X	X	X	–
Involvement of the CPA	+ CPA	±	±	X	±	X

^a^Extension through the modiolus into the internal auditory canal

^b^Extension through the macula cribrosa into the internal auditory canal

^c^Extension through the modiolus and the macula cribrosa into the internal auditory canal. For intravestibulocochlear tumors and location in the fundus of the IAC, the differentiation to transmacular and transmodiolar extension might be difficult. In our case series no tumor with certain transmodiolar and transmacular (i.e., translabyrinthine) extension was present. Also, this type of tumor extension is not part of the Kennedy et al. classification [21]

*IAC* internal auditory canal, *CPA* cerebellopontine angle, *SSC* semicircular canals

Here, we report our experiences with the surgical management of cochleovestibular schwannomas with TMOD or TMAC extension and hearing results after CI.

## Methods

For this retrospective analysis, patients with a TMOD, TMAC, TLab, or transotic (TO) extension of cochleovestibular schwannoma were included (classification after [21, 54]; Table 1). Patients were selected from a consecutive personal case series of the first author at a tertiary (university) referral center with an interdisciplinary skull base center. The tumors were either primary ILS or classical (intrameatal) schwannomas with secondary invasion of the inner ear and with or without extension into the CPA. Patients were treated between November 2013 and April 2020 and underwent either complete or partial tumor removal with or without CI.

Depending on tumor location and extension, surgery involved a translabyrinthine/transotic approach to the IAC and the CPA or a transmeatal partial or subtotal cochleoectomy (for details see Table 2 and “Results”; [2, 33, 35, 36, 38, 39]).

**Table 2:** Demographic and baseline data, management and postoperative audiological data with cochlear implant

No.	Age^a^	m/f	Side R/L	Pre-op. 4PTA (dB HL)^b^/ WRS_max_ (%)		DHL^c^ (years)	Tumor localization/extension	Surgery, CI electrode type, Fig. no.	Residual tumor?	Post-op./last WRS_65_ numbers/monosyllables [%] (months) with CI
				Ipsi	Contra					
1	52	m	R	>110/0	8.75/100	22	Transotic + CPA (inner ear, middle ear, IAC, CPA)	Translabyrinthine and transotic approach to IAC and CPA, removal of posterior canal wall, blind sack-like closure of external auditory canal Fig. 2a,b	No	N/A
2	65	f	R	93.75/0	>110/0 (without contralateral CI)	Ca. 28	Transmodiolar + CPA	Translabyrinthine and transmeatal transotic approach to IAC and CPA Fig. 2c,d	No	N/A
3	45	f	R	50/95^d^	13.75/100	5	Transmodiolar	Partial cochleoectomy (extended double cochleostomy) and push-through technique CI: Synchrony, Mi1200 FLEXSOFT Fig. 2e,f and 7a	Modiolus/Fundus of IAC	50/15 (22)
4	48	f	R	>110/0	18.75/100	N/A	Transmodiolar + vestibule; 3 years before: retrosigmoid tumor resection (intrameatal tumor parts)	Partial cochleoectomy and tumor removal via pull-through technique + labyrinthectomy CI: CI512 Fig. 3	Modiolus	100/65 (36)
5	28	m	R	>110/0	>110/0	~5	Transmodiolar + CPA (secondary invasion of the inner ear), NF2; 4 years before: partial tumor removal via retrosigmoid approach; complete removal of contralateral tumor with deafness	Partial cochleoectomy and push-through technique CI: CI512 Fig. 4a,b and video online via QR code above	Modiolus, IAC, CPA (large)	100/85 (24)
6	62	f	R	>110/0	20/100	~10	Transmodiolar + vestibule	Subtotal cochleoectomy and labyrinthectomy CI: CI512 Fig. 4c,d and 7b	Modiolus/Fundus/IAC	0/0 (24)
7	56	f	L	>110/0	10/100	>20	Transmodiolar + vestibule	Subtotal cochleoectomy and stapedectomy CI: Synchrony CMD Fig. 4e,f	Fundus (small)	100/65 (6)
8	23	m	R	47.5/95^e^	8.75/100	<1	Transmacular	Translabyrinthine approach to IAC CI: CI612 Fig. 5	No	100/70 (1)
9	24	f	R	33.75/100^e^	11.25/100	~0.3	Transmacular	Translabyrinthine approach to IAC (CI at second stage)	No	N/A
*N* *=* *9*	*45* *±* *16*	*3m/6f*	*8R/1L*	*92*^*e*^*/32* *±37/48*	*37/78* *±47/44*	*11* *±* *11*	*–*	*–*	*–*	*75/50* *±* *42/34*

^a^Age at surgery in years

^b^In cases of anacusis (no measurable threshold, >110 dB HL), PTA was set at a dummy code of 120 dB HL (Plontke et al. [32])

^*c*^*DHL* duration of hearing loss in the side of the tumor

^d^Patient 3: progressive hearing loss over 5 years, WRS_65L_ 5%, WRS_max_ 95% (hearing aid could not be used because of low uncomfortable loudness threshold), vertigo

^e^Patients 8 and 9: fluctuating hearing with secondary cochlear endolymphatic hydrops confirmed with magnetic resonance imaging (MRI) hydrops sequence (patient 8, Fig. 5c); patient 9: additional dizziness

*m/f* male/female, *R/L* right/left, *4PTA* air-conducted pure tone average (500, 1000, 2000, 4000 Hz in decibel hearing level, dB HL), *ipsi* ipsilateral, *contra* contralateral, *WRS*_*max*_ maximum percentage of monosyllables, *WRS*_*6*__5_ percentage of multisyllabic numbers and of monosyllables in quiet at 65 decibel sound pressure level, dB SPL, *CI* cochlear implant (CI512/612: Nucleus, Cochlear, Sydney, Australia; Synchrony: MED-EL, Innsbruck, Austria), *IAC* internal auditory canal, *CPA* cerebellopontine angle, *N/A* not available/not applicable, *Fig. no.* figure reference in this manuscript

Earlier audiological results for patient 4 were reported as patient 9 in Plontke et al. [36] and for patient 5 in Rahne et al. [39]

Selection of the type of cochlear implant and electrode array was based on experience and audiological results with previous surgery for ILS [35–38]. In addition, we ascribed to the hypothesis that a perimodiolar electrode array position with close contact of the electrode pads to the spiral ganglion cells in Rosenthal’s canal (here: CI512, CI612, CMD electrodes) with cartilage and fibrosis lateral to the array will lead to reduced spread of the electric field, contributing to a good hearing result after partial and subtotal cochleoectomy [56]. Furthermore, the improved magnetic resonance imaging (MRI) compatibility of the magnets in the receiver coil for easier MRI follow-up of the residual tumor was considered, which at that time was mainly present for a limited number of implant models (e.g., Synchrony FLEXSOFT, MED-EL, Innsbruck, Austria and Synchrony CMD, MED-EL, later also CI612, Cochlear, Sydney, Australia; Table 2).

Because of the small number and heterogeneity of patients with this rare tumor entity, statistical evaluation was mainly descriptive with a focus on surgical management and adverse events. In patients who underwent CI, the primary endpoint was word recognition in quiet conditions (Freiburger multisyllabic numbers and monosyllables at 65 dB SPL), with masking of the contralateral ear.

Secondary outcome parameters were intraoperative, electrically evoked (via the implant) compound action potentials (eCAP), impedance measures, the presence of a wave V in intraoperative and postoperative electrically evoked brainstem potentials (eABP), and characteristics during implant fitting and programming.

The eCAP were measured with the settings included in the respective manufacturer’s implant programming software (AutoNRT or AutoART). In patients who received a Nucleus implant, a transmodiolar stimulus was used for the eABP [11, 39]. The same stimulus was also used for recording of additional eCAP (AdvancedNRT). In patients with Synchrony implants, individual electrodes or electrode clusters distributed along the array were stimulated.

## Results

**Fig. 2:**
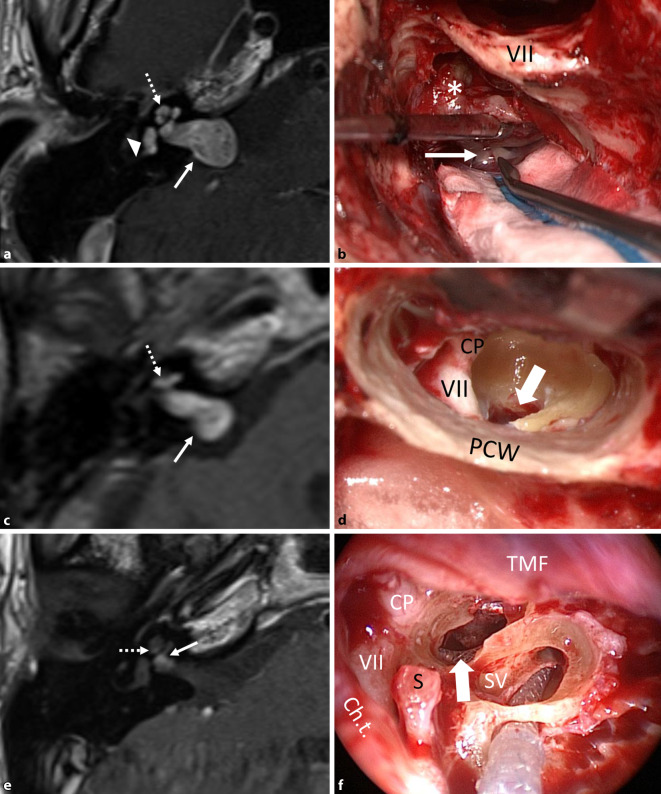
**a**,**b** Patient 1 in Table 2. **a** Magnetic resonance imaging (MRI, axial, T1-w + gadolinium, Gd) showing the transotic tumor with typical gadolinium enhancement in the entire inner ear, IAC and CPA (→), (middle ear extension not shown). **b** Intraoperative picture showing the facial nerve canal in the mastoid (VII) after lateral petrosectomy and tumor (*asterisk*) in the IAC + CPA during separation from the facial nerve (→). **c**,**d** Patient 2 in Table 2. **c** MRI (axial, T1-w + Gd) showing the tumor in the cochlea and in the IAC + CPA (→). **d** Intraoperative view of the fundus of the IAC (→) after combined translabyrinthine and transmeatal-transotic tumor resection. **e**,**f** Patient 3 in Table 2. **e** MRI (axial, T1-w + Gd) showing a transmodiolar ILS with tumor in the second turn of the cochlea and extension into the fundus of the IAC (→). **f** Intraoperative view after tumor resection from the cochlea with the implant electrode array at the lateral cochlear wall (→). The basal turn was free of tumor. *VII* facial nerve, *Ch.t.* chorda tympani, *CP* cochleariform process; *CPA* cerebellopontine angle, *Gd* gadolinium, *IAC* internal auditory canal, *PCW* posterior canal wall, *TMF* tympanomeatal flap, *S* stapes head, *SV* scala vestibuli, *w* weighted. *Dotted arrow *tumor in cochlea, *triangle* tumor in vestibule

**Fig. 3:**
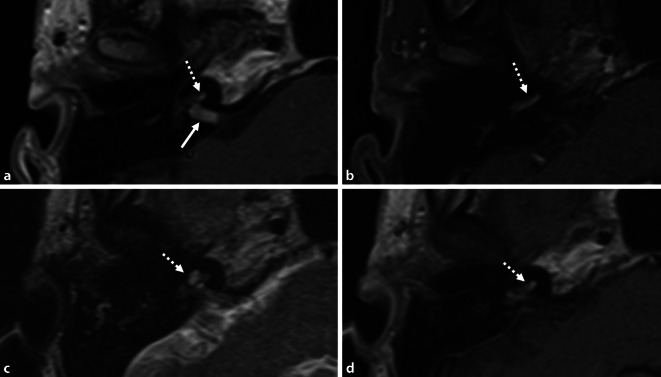
Patient 4 in Table 2. MRI (axial, T1‑w, + Gd). Transmodiolar ILS with resection of the intrameatal and, later, the intracochlear tumor parts. **a**,**b** Schwannoma in the IAC (→) and missed tumor in the cochlea (*dotted arrows*; 2013). **c**,**d** Situation 3 years after neurosurgical resection of the intrameatal and before resection of the intracochlear tumor parts

**Fig. 4:**
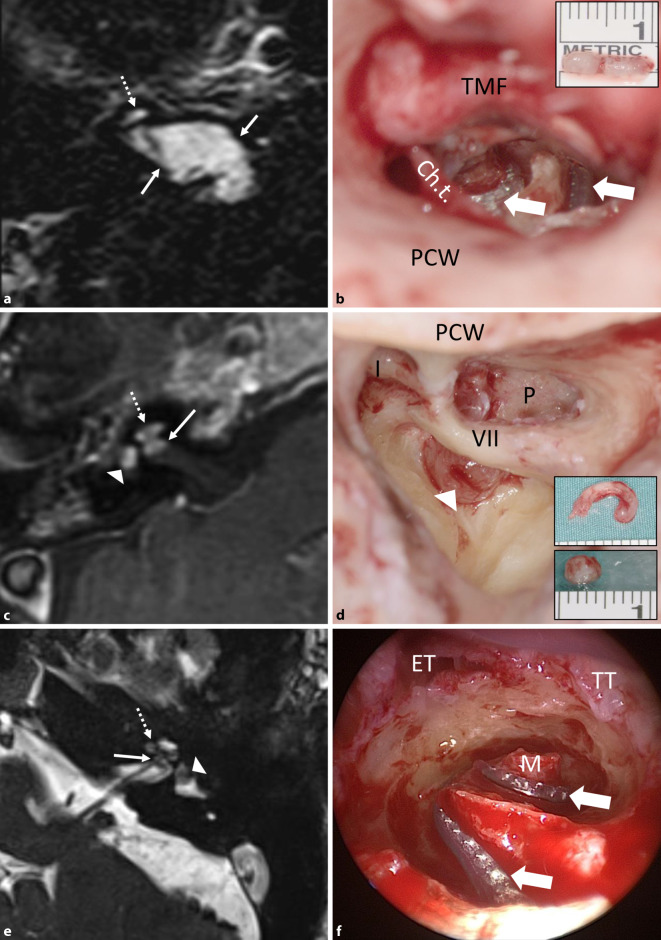
**a**,**b** Patient 5 in Table 2. **a** MRI (axial, T1-w + Gd) showing the schwannoma in the IAC + CPA (→) with secondary, transmodiolar extension in the cochlea in a patient with neurofibromatosis 2 after partial tumor resection via a retrosigmoid approach. The residual tumor was stable during a 4-year observation period. **b** Intraoperative view with the perimodiolar electrode array in the first and second cochlear coils after intracochlear tumor resection (insert picture). The modiolus was completely preserved (Rahne et al. [39]). The surgical technique is illustrated in the intraoperative video (2D, 3D). **c**,**d** Patient 6 in Table 2. **c** MRI (axial, T1-w + Gd) showing the tumor in the cochlea, in the vestibule, and in the fundus of the IAC. **d** Intraoperative view with tumor in the vestibule. Upper inset image: intracochlear tumor parts, removed through a transmeatal, subtotal cochleoectomy. Lower inset picture: intravestibular tumor parts. **e**,**f** Patient 7 in Table 2. **e** MRI (axial, T2-w) showing an intravestibulocochlear ILS with transmodiolar extension into the fundus of the IAC (→) as a missing fluid signal. **f** Intraoperative view with a perimodiolar implant electrode array (custom-made device, →) after tumor resection with preservation of the first and second turn modiolus. *VII* facial nerve, *I* incus body, *Ch.t.* chorda tympani, *ET* eustachian tube, *Gd* gadolinium, *IAC* internal auditory canal, *P* promontory; *PCW* posterior canal wall, *TMF* tympanomeatal flap, *TT* tensor tympani muscle, *w* weighted. *Dotted arrow* tumor in cochlea, *triangle* tumor in vestibule

**Fig. 5:**
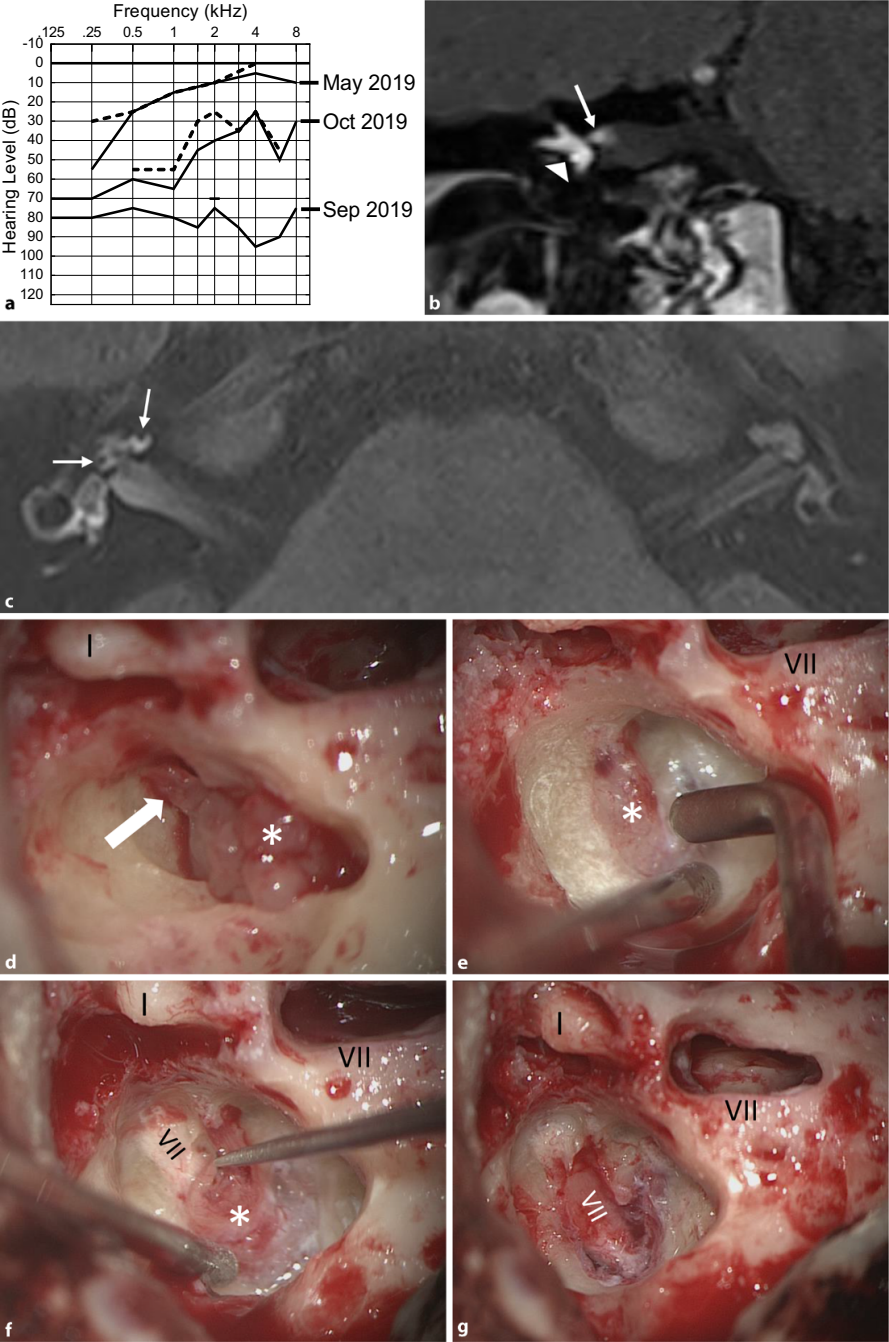
Transmacular ISL (patient 8 in Table 2). **a** Pure-tone audiogram showing fluctuating hearing loss (*full line*: air conduction, *dashed line*: bone conduction) most likely based on a secondary cochlear hydrops because of the tumor in the vestibule (**c**). **b** MRI (coronal, T1-w + Gd) showing the tumor in the right vestibule (*triangle*), the ampullary ends of the superior and lateral semicircular canals and extension along the superior vestibular nerve into the IAC (→). **c** MRI with hydrops sequence (3D inversion recovery, 6 h after systemic Gd application) showing a mild cochlear hydrops (→). **d** Intraoperative view with the tumor (*asterisk*) in the vestibule and along the superior vestibular nerve towards the fundus of the IAC (→). **e**–**g** The tumor (*asterisk*) is gradually separated from the facial nerve under facial nerve monitoring control. *VII* facial nerve, *Gd* gadolinium, *I* incus body, *w* weighted

From a total of 53 consecutive patients with ILS, 9 had tumors with TMOD (*n* = 6), TMAC (*n* = 2) or TO (*n* = 1) extension. Involvement of the CPA (+CPA) was found in three patients (Figs. 2, 3 and 4). Demographic and baseline data, tumor location, duration of hearing loss before surgery, surgical management, and if appropriate, hearing results with CI are shown in Table 2. All patients were informed of the different management options: 1) wait and test and scan, 2) radiotherapy, 3) complete or incomplete tumor removal, and 4) if possible, hearing rehabilitation with CI. None of the patients chose radiotherapy. Patient 3 was observed until the hearing threshold deteriorated, a hearing aid could not be tolerated because of a reduced uncomfortable loudness threshold, and symptoms of vertigo developed. Patient 5 with NF2 and bilateral deafness was observed until the ipsilateral residual tumor after partial tumor resection had remained stable for some years. The two patients with transmacular tumor extension (8 and 9) showed fluctuating hearing loss, likely because of a secondary endolymphatic cochlear hydrops detected on MRI (Fig. 5a, c). Patient 9 reported increasing dizziness.

Tumor removal was done by previously described surgical techniques either completely (patients 1, 2, 8, and 9; Fig. 2a–d and 5) or incompletely (patients 3–7; Fig. 2f, 3 and 4). Surgical removal of the TO tumor through a translabyrinthine/transotic approach and a lateral petrosectomy (patient 1; Fig. 2a, b) also has been described before [38]. In the two patients with TMAC extension from the vestibule along the superior vestibular nerve into the IAC, tumor removal was done via a translabyrinthine approach, preserving the cochlear nerve and the cochlea (Fig. 5d–h). Incomplete tumor removal was carried out by a transmeatal partial or subtotal cochleoectomy [33, 35, 36, 38] or via push-through or pull-through techniques (also called pipe cleaner, beach towel, or dental floss techniques) [2, 26, 36, 39]. The video (2D and 3D versions, see QR code) shows the transmeatal surgical removal of the intracochlear tumor parts through a partial cochleoectomy and CI in the patient with NF2.

Postoperatively, two patients (patients 7 and 9) reported temporary moderate vertigo. Another patient (patient 3) experienced continuation of a pre-existing vertigo for some weeks after surgery, but the condition continuously improved during the first postoperative year. In patient 2 a temporary incomplete facial paralysis (House-Brackmann II–III) developed but resolved completely within a couple of months. There were no severe adverse events.

Five patients received an implant with a perimodiolar electrode array, either with a Contour Advance electrode (Nucleus CI512, *n* = 3; Nucleus CI612, *n* = 1; Cochlear) or as a custom-made device (Synchrony Mi1200 CMD, MED-EL, *n* = 1 [37]). One patient received an implant with a lateral wall electrode array (Synchrony Mi1200 FLEXSOFT, MED-EL).

Intraoperatively, an increased impedance in one electrode was found in two patients. In all other patients, electrode impedances were homogeneous and below 15 kΩ. The intraoperative recording of eCAP (AutoNRT or AutoART) showed measurable thresholds for 1–5 electrodes in 3 patients. All electrodes showed measurable eCAP thresholds only in patient 8.

**Fig. 6:**
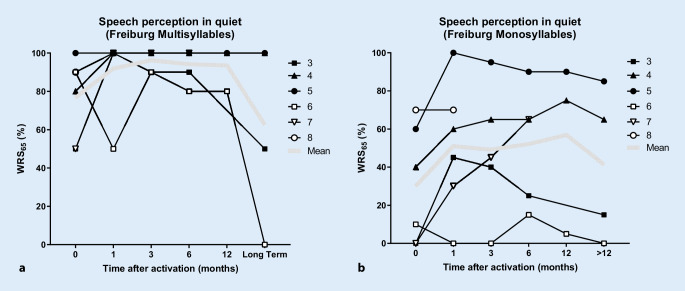
Results of speech audiometry testing for **a** multisyllables (numbers) and **b** monosyllables with the cochlear implant in quiet at 65 dB SPL (WRS_65_) as a function of time after activation of the speech processor

**Fig. 7:**
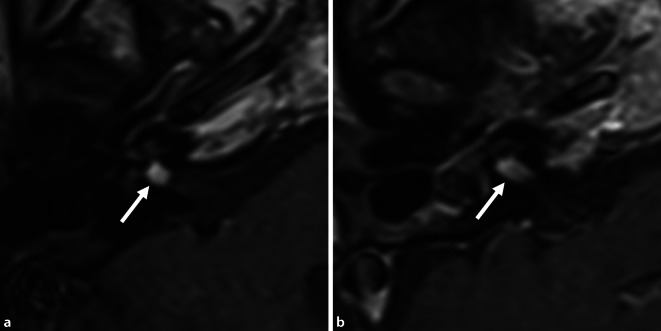
Postoperative MRI (axial, T1-w + Gd) for the two patients with poor hearing outcomes. **a** Patient 3, at 2 years after removal of the intracochlear tumor parts and CI. Imaging of the IAC and the inner ear was possible by placement of the receiver coil of the Synchrony implant at a distance of approximately 9 cm from the outer ear canal. The tumor part in the fundus of the IAC had increased in size only very slightly by 0.5 mm (compare preoperative MRI: Fig. 2e). **b** Patient 6, at 2 years and 5 months after removal of the intravestibulocochlear tumor parts and CI. Because of pain and heat development, the magnet of the CI512 model had to be temporarily removed from the patient under local anesthesia. The MRI showed growth of the tumor parts in the IAC by 3.6 mm (compare preoperative MRI: Fig. 4c). There was no tumor recurrence in the inner ear in the two patients. *CI* cochlear implant, *w* weighted, → tumor in the IAC

Postoperative audiological results with the implant are listed in Table 2 (last available measurement) and are shown individually over time in Fig. 6. All patients had good word recognition scores at 65 dB (WRS_65_) for multisyllabic numbers in quiet conditions (80–100% at 65 dB SPL, not later than 12 months after first fitting). Of the six patients who underwent CI, four reached good to very good WRS_65_ for monosyllables (65–85% at 65 dB SPL). Two patients had only poor WRS_65_ after 6 months (patient 3: 25%, and patient 6: 15% at 65 dB SPL), with further worsening of results thereafter. The follow-up MRIs in these two patients showed only minimal tumor growth of 0.5 mm (2 years after implantation) in patient 3 (Fig. 7a), and growth of 3.6 mm (2 years and 5 months after implantation) in patient 6 (Fig. 7b).

On average, patient WRS_65_ values were 94 ± 9% for numbers and 52 ± 31% for monosyllables at 65 dB SPL (after 6 months, *n* = 5). Twelve months after the first fitting of the audio processor, data were available for three patients (patients 4, 5, and 6), and the WRS_65_ was 93 ± 12% for numbers and 57 ± 45% for monosyllables.

Postoperative impedances were unstable in four patients and varied over time. In patient 5, a functional short circuit between two electrodes was found after 6 months. The other patients had stable, homogeneous impedances below 15 kΩ.

The eABP data were available for four patients. In three patients with a Nucleus CIx12 implant, a wave V could be recorded at thresholds of 160–180 current units (cu). In two patients, this correlated with eCAP thresholds that were recorded with the same stimulus. In patient 7, who had the Synchrony CMD implant, no eABP thresholds could be measured.

Postoperative implant programming could be done in all patients based on subjective information through loudness scaling. In 3 patients, 2–4 electrodes had to be deactivated because of discomfort or missing loudness growth. In four patients, relatively high charge levels with an increased pulse width were necessary.

## Discussion

This case series presents our experiences with the surgical management of a rare subgroup of cochleovestibular schwannomas, including hearing rehabilitation with cochlear implant in some of these patients. In two patients with TO or TMOD tumor extension including the CPA (patient 1, Fig. 2a, b, and patient 2, Fig. 2c, d), complete tumor removal including the intramodiolar tumor parts had priority for the patients over hearing rehabilitation; thus, CI was not possible. In the two patients (8 and 9) with TMAC tumor growth, the tumor could be completely removed through a translabyrinthine approach with preservation of the cochlear and facial nerves (Fig. 5). Already at the first fitting, the patient with TMAC who underwent CI had reached surprisingly good WRS_65_ for monosyllables (70% at 65 dB; Table 2, Fig. 6). The five patients with incomplete tumor removal showed a larger variance in WRS_65_ for monosyllables (minimum 0%, maximum: 100%; Fig. 6). The mean WRS_65_ for monosyllables after 6 months (52%) was still similar to results for other patients having CI [18, 24, 43], especially compared to patients with implants for single-sided deafness [1, 13, 16, 40, 55]. These results are also similar to or better than those reported in small case series with CI after translabyrinthine resection of classical VS (IAC ± CPA) [4, 17, 22, 42, 57]. The WRS_65_ values, however, were not as good and consistent as those reported for patients with CI after removal of solely intracochlear and intravestibulocochlear schwannomas [35, 36, 56]. There were no serious adverse events.

Intraoperative eCAP and intraoperative and/or postoperative eABP were recorded as secondary outcome parameters. Although for preoperative eABP measurements only the promontory can be stimulated, the intracochlear implant electrode was used as an optimal stimulation site for intraoperative and postoperative recordings. The recordings in patient 7 (CMD electrode) were performed together with the manufacturer. No wave V could be detected in this patient, most likely because of suboptimal stimulation parameters. In all other patients who had a CI, a clear wave V could be recorded. Although all patients (including patient 7) achieved an auditory impression, word recognition ranged from very good (patient 5) to very poor (patient 6). Thus, eABP measurements in principle correlated with implant function, but without a correlation with the expected word recognition. Similar conclusions can be made for the eCAP with preset stimulation parameters, which could be recorded for all electrodes in only one patient.

These observations also correlated with the CI programming. Electrodes had to be deactivated in 3 out of 6 patients, and charge levels had to be set relatively high in 4 out of 6 patients. The fluctuation impedances indicate intracochlear changes, which might explain the deterioration in speech understanding that some of the patients experienced. Whether these changes are the result of increasing fibrosis or residual tumor tissue could not be determined.

The limitations of the presented observations lie in the small number and heterogeneity of the patient group and the relatively short follow-up period (maximum 36 months). The ILS are rare tumors, and the patients in this case series are a subgroup, representing only 17% (9 out of 53) of our overall cohort of ILS patients. Other authors have reported this subgroup of tumor locations and extensions (TMOD, TMAC, TO, and TLab) in 8% [54], 17% [10], 29% [21], and 42% [44] of their respective case series.

We note that in an individual case, it often cannot be determined whether the tumor is a primary ILS (arising from the inner ear) or started from the IAC with secondary invasion into the inner ear (invasive schwannoma [27]). Initial imaging results frequently already show the tumor in both the inner ear and the fundus of the IAC. An exception in our case series was the patient with NF2 (patient 5). Initial MRI showed the tumor in the IAC and the CPA but not in the inner ear (suggesting secondary ILS or invasive schwannoma). In patients 2, 3, and 4, the origin of the tumor was assumed to be in the IAC. In view of missing earlier MRI images, however, we could not be certain. In addition, a differentiation between TLab and TMOD/TMAC tumor extension is often difficult if the tumor is located in the cochlea and the vestibule (± semicircular canals) and completely fills the fundus of the IAC (e.g., patient 6, Fig. 4c). For surgical management, however, the differentiation between primary versus secondary and TMOD versus TLab does not play a significant role. In cases of tumor extension through the modiolus, hearing rehabilitation with a CI is not compatible with complete tumor removal because spiral ganglion cells in the modiolus are necessary for electrical stimulation. The TMAC tumors represent a special situation, at least with limited extension into the IAC. In these cases, complete tumor removal with preservation of the cochlear nerve in the IAC and very good results for speech understanding with implants seem to be possible (patient 8; Figs. 5 and 6, Table 1).

In addition to surgery, radiotherapy is another treatment option; however, the proximity of the sensory or ganglion cells to the target region needs to be considered. Radiotherapy arrests the tumor growth of classical (IAC ± CPA) VS. Initial reports for radiotherapy for VS (no ILS) and CI showed positive results for hearing rehabilitation [5, 9, 31]. Although data for the tolerance doses of the various inner ear structures are sparse, even the frequently recommended cochlear tolerance doses of approximately 5 Gy for single-fraction radiosurgery (SRS) and 35 Gy for fractionated stereotactic radiotherapy (FSRT) are difficult to achieve in any inner ear location, given the required therapeutic doses of approximately 12 Gy in SRS and 54 Gy in FSRT [30]. Irrespective of the radiotherapy technology (SRS via gammaknife or cyberknife, FSRT via linear accelerator), deterioration of existing hearing ability (e.g. cases #3, #8, #9) and restrictions for later rehabilitation with CI are expected.

Even a CI without a therapeutic intervention with respect to the tumor (implant + wait and test and scan) appears to be possible. The first results in patients with intrameatal tumors (± CPA) were promising [5]. In patients with a tumor in the inner ear, however, the implant electrode array would need to be inserted through the tumor, as described by Carlson et al. [6].

After gross total tumor resection (patients 1, 2, 8, and 9 in Table 1), recommendations for MRI follow-up are similar to those for resection of intrameatal (± CPA) tumors. No specific recommendations exist for patients with possible tumor remnants in the modiolus (e.g., after resection of intracochlear, intravestibular, and intravestibulocochlear schwannomas through a subtotal cochleoectomy [35, 36]) and/or labyrinthectomy or with only small remnants in the modiolus (e.g., patient 4) or in the fundus of the IAC (e.g., patient 7). Any presence of residual tumor bears the significant risk of growth of the remaining tumor tissue. Even after gross total resection, however, long-term follow-up with MRI is necessary, which patients explicitly need to be told. In a retrospective study, 52 out of 396 (13%) patients with gross total tumor resection experienced a recurrence after a mean of 7.5 years [29]. In contrast, after incomplete but near total resection of intrameatal tumors (± CPA), most patients experienced no or no relevant tumor recurrence. In a series of 1143 patients with translabyrinthine removal of VS evaluated intraoperatively as total (*n* = 978), near total (*n* = 140), or subtotal (*n* = 25) tumor removal, Hahn et al. (2013) reported a rate of revision surgery of 1.2%, i.e., 14 patients with 2 initially receiving total, 5 near total, and 6 subtotal excisions. The authors concluded that most residual tumors disappear spontaneously, probably due to devascularization [15]. Other groups have reported similar observations. The authors noted that the growth rate of residual tumors is less for near total than for subtotal resection and that long-term MRI follow-up (~10 years) is necessary [7, 19, 20, 48].

Imaging follow-up is more difficult for patients with CI. First, the magnet in the receiver coil leads to significant artefacts, which can impede the evaluation of the inner ear, the IAC, and the CPA. This problem can be counteracted by placement of the receiver coil at a distance of about 9–10 cm from the outer ear canal, which enables imaging of the inner ear and the IAC/CPA despite the implant (Fig. 7a; [46, 49, 51]). Second, the magnetic field can induce significant heat and force, which may lead to pain and magnet dislocation, necessitating revision surgery [14, 23, 47, 49]. Temporary magnet removal or correct securing of a head splint can be used to overcome these problems. For the latter, standard operating procedures are available at our and many other institutions. Recently, magnets have been implemented in some implant models that align with the magnetic field, facilitating MRI follow-up [52, 53].

## Conclusion

Cochleovestibular schwannomas with transmodiolar or transmacular extension are very rare and represent a special entity with respect to management. A therapeutic approach including incomplete and complete tumor removal requires a differentiated assessment considering the therapeutic goal and tumor location and extension. In addition, in these patients, CI can be an option for hearing rehabilitation.

## Caption Electronic Supplementary Material

Video 1: the video (2D and 3D versions) demonstrates the surgical technique for removal of the intracochlear tumor parts, cochlear implantation, and cochlear defect reconstruction in a patient with NF2 (patient 5, Fig. 4a,b). After mastoidectomy, posterior tympanotomy, drilling of the implant bed, and canaloplasty for a better approach to the cochlea, the tumor was exposed in the first and second turns of the cochlea by a transmeatal approach. Parts of the tumor were microsurgically removed through the openings of the cochlear capsule. Intrascalar tumor remnants medial to the modiolus were pushed through with a shortened insertion test device (MED-EL, Innsbruck, Austria). Insertion of the implant electrode array was done as in standard implant surgery, i.e., through the posterior tympanotomy and the extended round window. The electrode was additionally approximated towards the modiolus and thus the cochlear spiral ganglion cells in Rosenthal’s canal by means of small cartilage chips. The cochlear defect was closed with a cartilage-perichondrium compound transplant from the cymba conchae, soft tissue, and fibrin glue. A silicon foil was inserted into the middle ear cavity to prevent adhesions between the posterior part of the eardrum (reinforced with temporalis muscle fascia) and the medial wall of the middle ear. Outer ear canal silicon foils and dressing were removed after 4 weeks. (The surgical videos (2D and 3D) were recorded with a fully digital microscope (ARRISCOPE). We thank Dr. Armin Schneider, Munich Surgical Imaging GmbH (formerly ARRI Medical GmbH), Munich, Germany, for support with cutting and postproduction.)

Video 2 (3D version): the same as video 1 but in a 3D version. It can be viewed with a Smartphone 3D Cardboard or a 3D screen.
